# Ethical Dilemmas in Postnatal Treatment of Severe Congenital Hydrocephalus

**DOI:** 10.1017/S0963180115000316

**Published:** 2016-01

**Authors:** DOMINIC WILKINSON

**Keywords:** hydrocephalus, children, medical ethics, withdrawing treatment, medical futility, neonatal intensive care

## Abstract

Severe congenital hydrocephalus manifests as accumulation of a large amount of excess fluid in the brain. It is a paradigmatic example of a condition in which diagnosis is relatively straightforward and long-term survival is usually associated with severe disability. It might be thought that, should parents agree, palliative care and limitation of treatment would be clearly permissible on the basis of the best interests of the infant. However, severe congenital hydrocephalus illustrates some of the neuroethical challenges in pediatrics. The permissibility of withholding or withdrawing treatment is limited by uncertainty in prognosis and the possibility of “palliative harm.” Conversely, although there are some situations in which treatment is contrary to the interests of the child, or unreasonable on the grounds of limited resources, acute surgical treatment of hydrocephalus rarely falls into that category.

## Introduction

Ethical questions arising in the care of children with congenital or acquired severe brain injury have been debated for more than 40 years.^1^ As noted elsewhere in this issue, decisions about treatment for such children give rise to some of the most challenging neuroethical dilemmas in pediatrics.^2^ Questions about whether or not certain types of treatment are in the best interests of the child, and whether parents are permitted to request that a particular treatment be withheld or provided, are central to the debate.^3^

## Severe Congenital Hydrocephalus

The human brain has a set of central fluid reservoirs called the ventricles. These reservoirs normally contain a small amount of cerebrospinal fluid (CSF), which circulates through tiny channels and flows over the outside of the brain and spinal cord.^4^ Congenital enlargement of the ventricles (ventriculomegaly or hydrocephalus) is seen in approximately 5 in 10,000 live births.^5^ There are a number of different causes. In some cases, severe congenital hydrocephalus (SCH) arises from blockage of the normal flow of CSF, for example, because one of the drainage channels is narrowed. In other cases, the ventricles are enlarged because of damage to and atrophy of the surrounding brain, for example, because of infection or infarction.^6^ In the current era, 80 percent of cases of congenital hydrocephalus are diagnosed antenatally by ultrasound.^7^

Ventriculomegaly can be seen in isolation; alternatively, it may be identified in association with abnormalities of the brain or other organs.^8^ The size of ventricles before birth is often used to classify severity, with ventricles larger than 15 mm typically labeled “severe” ventriculomegaly/hydrocephalus.^9^ At the most severe end of the spectrum is a condition called hydranencephaly, in which all, or almost all, of the cerebral hemispheres are replaced by fluid.^10^

The outcome for infants with mild or moderate degrees of ventriculomegaly is variable; most do not have significant long-term neurological problems.^11^ In contrast, infants with severe hydrocephalus have a high rate of significant long-term disability. Only 5–8 percent have a normal neurodevelopmental outcome.^12^

There are various surgical treatments for hydrocephalus. Principal among these is insertion of a shunt, a tube to relieve the buildup of CSF and divert it (usually into the abdomen). The shunt insertion procedure is relatively low risk.^13^ However, there is a moderately high rate of complications over time. More than half of shunts block at least once over a ten-year period, and revision or reinsertion of shunts is more complicated.^14^ Approximately one in five children will develop an infection related to their shunt, a serious condition potentially requiring prolonged hospitalization and repeat surgery.^15^

Following prenatal diagnosis of SCH, a large proportion of women choose to terminate their pregnancy.^16^ However, many fetuses are diagnosed with SCH relatively late in pregnancy.^17^ Although in the UK termination of pregnancy is legally available in the third trimester (on the basis of a “substantial risk of serious handicap”),^18^ in other jurisdictions termination may not be an option. Where a pregnancy is continuing, in the setting of SCH obstetricians and women may elect to focus obstetric care on maternal well-being rather than fetal survival. If the fetal head is grossly enlarged because of hydrocephalus, alternatives include caesarean section (because of the difficulty of vaginal delivery) or cephalocentesis (needle drainage of fluid in the fetus’s brain immediately before delivery, usually leading to stillbirth).^19^

The ethical challenges around obstetric care following prenatal diagnosis have been described elsewhere.^20^ However, there is also potential for significant ethical dilemmas postnatally. Consider the following hypothetical cases.^21^

## Cases

### Case 1

Andrew is a 36-week-gestation newborn infant who was diagnosed on a 28-week ultrasound with severe fetal ventriculomegaly (lateral ventricles 19 mm bilaterally). His parents had been counseled before birth that he had a high chance of long-term disability. Termination of pregnancy had been discussed but was declined on religious grounds. However, Andrew’s parents have requested palliative care after birth. They do not wish to put him through surgical treatment for his hydrocephalus.

### Case 2

Bianca was born prematurely at 28 weeks gestation following a severe placental abruption. She needed resuscitation and emergency blood transfusion at delivery and was admitted to intensive care. Subsequently, Bianca showed evidence of severe brain damage from lack of oxygen. Brain scans showed a very large intraventricular hemorrhage (bleeding into the ventricles) with dilated fluid spaces. Over the ensuing weeks Bianca remained completely dependent on the ventilator for respiratory support. She showed evidence of hydrocephalus, with increasing head circumference and a bulging fontanelle. She had a persistently abnormal conscious state, with decorticate responses to painful stimuli. Bianca’s parents have rejected any suggestion of withdrawal or limitation of treatment. They have requested surgical intervention for her hydrocephalus and tracheostomy for long-term ventilation.

### Case 3

Carl was born at term following a normal pregnancy. He had a cranial ultrasound performed after birth because of poor feeding and concern about some unusual movements. The ultrasound revealed the presence of hydranencephaly. His parents were counseled about this condition, and it was explained that it was likely that he would die in infancy or be profoundly disabled if he survived into childhood. Carl remained stable, and his only medical treatment was provision of nasogastric feeding. His parents researched Carl’s condition on the Internet and have requested insertion of a ventriculoperitoneal shunt.

## Ethics and Nontreatment of Severe Hydrocephalus

In cases like that of Andrew (Case 1), there are a series of questions that we might ask. First, what is the likely outcome for him with treatment?

This first question raises an epistemic challenge. In some series, two-thirds of pregnancies are terminated,^22^ leaving only a small number of live-born infants to provide data on the outcome.^23^ Those infants who are born alive may not be representative of the larger group of fetuses with SCH (e.g., there is a higher rate of termination in the presence of other abnormalities). In one series from Ireland, where termination of pregnancy is not legal, out of 17 fetuses with apparently isolated SCH, 7 died (mostly in the setting of cephalocentesis).^24^ Out of the 10 surviving children, 5 had severe developmental delay and/or cerebral palsy, 4 had milder degrees of impairment (e.g., mild or moderate delay in learning to walk or talk), and 1 was developing normally at 4 years of age. These data are useful; however, given the small numbers there is necessarily significant uncertainty about the true rate of severe disability.^25^ Furthermore, the heterogeneity of the causes and course of prenatally diagnosed SCH makes it challenging to apply published figures to an individual case.

The second question is clearly normatively challenging. Is the level and probability of disability in children with SCH severe enough and high enough that nontreatment is consistent with Andrew’s best interests?^26^ It is extremely challenging to determine where the threshold should be for permissible limitation of treatment for newborn infants.^27^ One approach to answering this question appeals to normative criteria. For example, limitation of treatment would be permissible if there is clear and convincing evidence (more than a 50 percent chance) of severe disability such that the child has significant risk of a life not worth living.^28^

Do children with SCH risk a life not worth living? There is limited data on the quality of life of children with SCH. One Canadian study provides some relevant data, albeit from a mixed group of children with different degrees and causes of hydrocephalus.^29^ The study asked caregivers of children attending three neurosurgical clinics to complete a validated questionnaire assessing health-related quality of life. The majority rated their child’s quality of life positively. Seventeen caregivers (5%) returned responses indicating a health utility of < 0 (i.e., potentially equivalent to a life not worth living).^30^ Even if the preceding normative criteria are correct (and they may not be), it is difficult to know whether SCH meets them.

There is a further practical question to ask that is relevant to Andrew. If a shunt is *not* performed, what would his outcome be? Answering that question also poses an epistemic challenge, because in the current era almost all children with hydrocephalus receive surgical treatment.^31^ There are case reports (e.g., from countries or healthcare settings with limited access to neurosurgery) of untreated children surviving infancy, who develop a massive enlargement of their head.^32^ Data from an earlier period provide some insights. One series from Chicago in the 1950s described 45 infants with SCH judged to have such poor prognosis that surgery was not performed.^33^ Twenty-one children died (at an average age of three years), whereas 24 survived. Nine of the surviving children (followed for an average of eight years) were found to have severe physical and cognitive impairment, whereas another nine were judged to have “near-normal” function. These striking results highlight the prognostic uncertainty noted previously—surviving infants with severe hydrocephalus do not necessarily have severe disability. However, they also highlight the possibility of what could be called “palliative harm.” Infants who are untreated do not necessarily die in the newborn period. Without active treatment, infants may survive for years, or indeed into adult life. However, as a consequence of not being actively treated, those children risk surviving in a worse state—with a greater degree of physical, cognitive, or sensory disability, or with other complications of their distended ventricles and enlarged head.^34^

## Ethics and Treatment of Severe Hydrocephalus

I have described serious potential ethical challenges for nontreatment in some cases of SCH. In contrast, Cases 2 and 3 represent situations in which clinicians might feel ethical qualms about *provision* of treatment. Bianca’s clinical picture—severe hypoxic brain injury and hydrocephalus—would make severe cognitive and motor disability virtually inevitable if she survives.^35^ Surviving children with hydranencephaly all have profound cognitive impairment.^36^ Some clinicians might regard treatment as futile in either or both of these cases and could refuse to provide surgery.^37^ Yet there are significant challenges in determining whether treatment for Bianca or Carl is futile. The concept of medical futility has come under sustained criticism.^38^ There are multiple different competing definitions of futility, all of which contain value judgements—about the appropriate goal of treatment and about the point at which the level or chance of benefit is sufficiently low that treatment should not be provided.

Fundamentally, there are only two ethical justifications for declining to provide treatment in Cases 2 or 3. Treatment should not be provided if it would be harmful to the child, or if it would be harmful to others (e.g., because of use of limited resources).^39^

Would neurosurgical treatment be harmful to Bianca or Carl? In Bianca’s case, the clinical team might be concerned about the possibility of medical treatment causing her to suffer. A recent UK court case concerning an infant referred to as “ZT” involved a very similar situation: an infant with hypoxic brain injury and hydrocephalus.^40^ Expert medical professionals gave evidence of ZT’s response to tracheal suction, required multiple times a day, and expressed concern that he was experiencing pain from this procedure. However, there are three arguments that undermine this concern about harm. First, those infants who are most severely affected may have little or no apparent response to pain. In ZT’s case, other medical testimony pointed to his lack of responsiveness to pain or deep stimulation as evidence of the severity of his brain damage.^41^ For Carl, with hydranencephaly, the absence of any cerebral cortex might be thought to be incompatible with pain perception.^42^ Paradoxically then, the greater the severity of hydrocephalus, the more tolerable treatment may be, and the less we may have reason to fear harm to the child.^43^ Second, a concern about suffering for the child is, or should be, something that intensive care consultants, anesthetists, or pain specialists should be able to address. Discomfort from tracheal suction is a very familiar problem in intensive care, one that neonatologists and other medical professionals have considerable experience in managing. Third, it might be the case that medical treatments cause ongoing symptoms of pain or discomfort for Bianca. Importantly, though, those symptoms are not likely to be directly related to treatment for her hydrocephalus. Shunt surgery is a relatively small surgical procedure and not usually particularly painful. Indeed, surgical treatment of her hydrocephalus might well reduce symptoms related to raised intracranial pressure. Concern about harm from medical treatment might give reasons not to continue invasive and unpleasant treatments such as prolonged mechanical ventilation for Bianca. It wouldn’t necessarily provide a clear reason to decline surgical treatment of hydrocephalus in either Bianca’s or Carl’s case.^44^

Would treatment for Bianca or Carl harm other people? In public health systems with limited resources, provision of highly costly treatment may mean that other patients are unable to access treatment. Even if treatment were not harmful, this concern might provide a strong ethical rationale for not providing treatment.^45^ Yet it is unclear whether neurosurgery in such cases would be prohibitively expensive. The costs of neurosurgery for hydrocephalus range from US$10,000 for simple shunt insertion/revision to $70,000 for hospital admission associated with shunt infection.^46^ Based on published rates of shunt infection and mortality in hydrocephalus, we can roughly estimate surgical treatment to cost an average of $9,400 per year of life saved across childhood.^47^ Whether or not this is judged to be prohibitive will depend on the resources available and therefore the cost-effectiveness threshold used, whether quality of life is included in the assessment of benefit, and the value placed on survival in a state of severe disability. By way of illustration, if survival were judged to have a utility of 0.1 for Bianca or Carl,^48^ treatment would be regarded as cost-effective at a threshold of $94,000 per quality-adjusted life year (QALY). This level of cost might be judged reasonable.^49^ Furthermore, in Carl’s case, the acute costs of shunt insertion are likely to be relatively modest, and he does not appear to require other expensive or limited medical resources. The most significant cost for Bianca is her ongoing need for mechanical ventilation and intensive care. At a daily cost of more than $3,500, and with no apparent prospect for weaning from respiratory support, Bianca’s medical care over a year might well exceed $1,000,000.^50^ It may be that some healthcare systems are sufficiently well resourced that they can meet these costs as well as the healthcare needs of all other patients. However, that is not the case in most public healthcare systems.

## Conclusions

How, then, should clinicians resolve dilemmas like the ones described previously for children with SCH? A palliative approach to care may be ethically permissible for some infants with SCH, particularly in situations in which the hydrocephalus is accompanied by other major abnormalities. However, it is important that prognosis— including the degree of uncertainty about the outcome and the quality of life likely to result—be assessed carefully, on the basis of the best available data, and communicated clearly to parents. Acute neurosurgical intervention for raised intracranial pressure may be a component of good palliative care for the child, both providing symptom relief and reducing the possibility of palliative harm. It should not usually be withheld, except in the context of a dying child.

Conversely, a palliative approach to treatment might be ethically mandatory in some children with the most severe forms of congenital hydrocephalus. Concerns about harm to the child, or about unreasonable use of limited resources, could justifiably lead to treatment being withheld, even if it were desired by parents. Yet, I have argued that the focus in such cases should usually be on the harmful nature and cost of intensive care and mechanical ventilation (or other similar interventions) rather than on neurosurgery per se. This would not apply to children who do not require expensive and burdensome treatments, such as Carl.

How will advances in neuroscience impact on decisionmaking in children with SCH? Developments in neuroimaging—for example, using functional imaging or tractography—might make it possible to predict the degree and nature of long-term impairment with a greater degree of certainty. Alternatively, functional imaging or related technologies might make it possible to detect patterns of neural activity that would substantiate concerns about a child suffering.^51^ Although these possibilities are intriguing, the technical challenges are considerable, and it seems unlikely that they will be available any time soon.^52^ Sadly, however, such advances will not in any way resolve the fundamental normative challenges that are at the heart of these neuroethical dilemmas.

## Notes

1. ShawA. Dilemmas of “informed consent” in children. New England Journal of Medicine 1973;289(17):885–90426965610.1056/NEJM197310252891704; McCormickRA. To save or let die: The dilemma of modern medicine. JAMA 1974;229(2):172–64406917; DuffRS, CampbellAG. Moral and ethical dilemmas in the special-care nursery. New England Journal of Medicine 1973;289(17):890–4.472912010.1056/NEJM197310252891705

2. Gillett G. Ashley, two born as one, and the best interests of a child. *Cambridge Quarterly of Healthcare Ethics* 25;2016:22–37.

3. In this article I focus on treatment decisions in the newborn period. Although withdrawal or withholding of treatment from newborn infants with predicted disability remains controversial, there is general acceptance that it is permissible in at least some circumstances. WilkinsonD. Is it in the best interests of an intellectually disabled infant to die? Journal of Medical Ethics 2006;32(8):454–9.1687762410.1136/jme.2005.013508PMC2563380

4. McKechnieL, VasudevanC, LeveneM. Neonatal outcome of congenital ventriculomegaly. Seminars in Fetal and Neonatal Medicine 2012;17(5):301–7.2281938210.1016/j.siny.2012.06.001

5. GarneE, LoaneM, AddorMC, BoydPA, BarisicI, DolkH. Congenital hydrocephalus—prevalence, prenatal diagnosis and outcome of pregnancy in four European regions. European Journal of Paediatric Neurology 2010;14(2):150–5.1941048910.1016/j.ejpn.2009.03.005

6. See note 4, McKechnie et al. 2012.

7. See note 5, Garne et al. 2010.

8. One abnormality that is sometimes found in association with hydrocephalus is spina bifida, found in 27% of cases of hydrocephalus in California but in only 17% of cases of severe hydrocephalus in the UK. JengS, GuptaN, WrenschM, ZhaoS, WuYW. Prevalence of congenital hydrocephalus in California, 1991–2000. Pediatric Neurology 2011;45(2):67–712176394410.1016/j.pediatrneurol.2011.03.009; HannonT, TennantPW, RankinJ, RobsonSC. Epidemiology, natural history, progression, and postnatal outcome of severe fetal ventriculomegaly. Obstetrics and Gynecology 2012;120(6):1345–53.2316875910.1097/aog.0b013e3182732b53 Although the ethical considerations in infants with spina bifida overlap with those involved in infants with isolated hydrocephalus, there are also some differences in prognosis and the burden of treatment. For this article I consider the issues for the wider group of infants with SCH.

9. See note 4, McKechnie et al. 2012.

10. CecchettoG, MilaneseL, GiordanoR, VieroA, SumaV, ManaraR. Looking at the missing brain: Hydranencephaly case series and literature review. Pediatric Neurology 2013;48(2):152–8.2333701210.1016/j.pediatrneurol.2012.10.009

11. DevaseelanP, CardwellC, BellB, OngS. Prognosis of isolated mild to moderate fetal cerebral ventriculomegaly: A systematic review. Journal of Perinatal Medicine 2010;38(4):401–9.2029814910.1515/jpm.2010.048

12. See note 4, McKechnie et al. 2012. BreezeAC, AlexanderPM, MurdochEM, Missfelder-LobosHH, HackettGA, LeesCC. Obstetric and neonatal outcomes in severe fetal ventriculomegaly. Prenatal Diagnosis 2007;27(2):124–9.1715211510.1002/pd.1624

13. VinchonM, RekateH, KulkarniAV. Pediatric hydrocephalus outcomes: A review. Fluids and Barriers of the CNS 2012;9(1):18.2292545110.1186/2045-8118-9-18PMC3584674

14. See note 13, Vinchon et al. 2012.

15. PrusseitJ, SimonM, von der BrelieC, HeepA, MolitorE, VolzS, et al. Epidemiology, prevention and management of ventriculoperitoneal shunt infections in children. Pediatric Neurosurgery 2009;45(5):325–36.1990719510.1159/000257520

16. See note 5, Garne et al. 2010; note 8, Hannon et al. 2012.

17. See note 12, Breeze et al. 2007.

18. See note 8, Hannon et al. 2012.

19. ChervenakFA, McCulloughLB. Ethical dimensions of fetal neurology. Seminars in Fetal and Neonatal Medicine 2012;17(5):252–52269570410.1016/j.siny.2012.05.006; ChervenakFA, McCulloughLB. Nonaggressive obstetric management: An option for some fetal anomalies during the third trimester. JAMA 1989;261(23):3439–40.272448810.1001/jama.261.23.3439

20. See note 19, Chervenak, McCullough 1989 and 2012.

21. These cases are based on a composite of real cases encountered.

22. See note 8, Hannon et al. 2012.

23. WilkinsonD. The self-fulfilling prophecy in intensive care. Theoretical Medicine and Bioethics 2009;30(6):401–10.1994319310.1007/s11017-009-9120-6

24. KennellyMM, CooleySM, McParlandPJ. Natural history of apparently isolated severe fetal ventriculomegaly: Perinatal survival and neurodevelopmental outcome. Prenatal Diagnosis 2009;29(12):1135–40.1982148110.1002/pd.2378

25. The 95% confidence interval for the rate of severe disability in live-born infants with severe SCH ranges from approximately 25% to 75%. AgrestiA, CoullBA. Approximate is better than “exact” for interval estimation of binomial proportions. American Statistician 1998;52(2):119–26.

26. As other articles in this issue note, Andrew’s interests are clearly entwined with those around him, and any assessment of his best interests should take into account the context in which he lives and the effects of treatment on his caregivers. See note 2, Gillett 2016. Bowyer L. The ethical grounds for the best interests of the child. *Cambridge Quarterly of Healthcare Ethics* 2016;25:63–9. For this article I have set aside the interests of the family and caregivers.

27. WilkinsonD. A life worth giving: The threshold for permissible withdrawal of treatment from disabled newborn infants. American Journal of Bioethics 2010;11(2):20–322133727310.1080/15265161.2010.540060PMC3082774; WilkinsonD. Death or Disability? The Carmentis Machine and Treatment Decisions for Critically Ill Children. Oxford: Oxford University Press; 2013.

28. See note 27, Wilkinson 2013, at 261–301.

29. KulkarniAV, CochraneDD, McNeelyPD, ShamsI. Medical, social, and economic factors associated with health-related quality of life in Canadian children with hydrocephalus. Journal of Pediatrics 2008;153(5):689–95.1857167210.1016/j.jpeds.2008.04.068

30. See note 29, Kulkarni et al. 2008. There are significant limitations to the use of data of this kind for evaluating whether or not a child’s life is worth living. See note 27, Wilkinson 2013, at 184–94. It is also important and instructive to note that socioeconomic factors including family income and parental education had a significant impact on health utility scores in this study. Furthermore, the limited data that are available provide no information on the quality of life of caregivers or other family members, factors that may influence caregivers’ assessment of the child’s quality of life, and that may be of relevance to an assessment of the child’s best interests. See note 26, Bowyer 2016.

31. ChiJH, FullertonHJ, GuptaN. Time trends and demographics of deaths from congenital hydrocephalus in children in the United States: National Center for Health Statistics data, 1979 to 1998. Journal of Neurosurgery 2005;103(2 Suppl):113–18.1637027510.3171/ped.2005.103.2.0113

32. MoghtaderiA, Rahimi-MovagharV, SafdariM. Spontaneous brain rupture: A complication of untreated hydrocephalus. Clinical Neurology and Neurosurgery 2005;108(1):48–511631114610.1016/j.clineuro.2004.10.015; SalvadorSF, HenriquesJC, MunguambeM, VazRM, BarrosHP. Hydrocephalus in children less than 1 year of age in northern Mozambique. Surgical Neurology International 2014;5:175.2559375910.4103/2152-7806.146489PMC4287916

33. YashonD, JaneJA, SugarO. The course of severe untreated infantile hydrocephalus: Prognostic significance of the cerebral mantle. Journal of Neurosurgery 1965;23(5):509–16, at 510.529526810.3171/jns.1965.23.5.0509 Surgery was not performed on those who exhibited “thin mantles, large heads, or generally unimposing appearance”

34. Palliative harm is a possibility wherever palliative care would be permissible on the grounds of poor prognosis but long-term survival is a possibility. It is particularly a problem when life-sustaining treatments are limited in incompetent patients on the basis of neurological prognosis. Other examples include patients who have respiratory support withheld or withdrawn and sustain a period of hypoxia but nevertheless survive, or patients who have artificial nutrition withheld but sustain enough oral intake to survive (in a malnourished state). Furthermore, palliative harm may lead to additional harm and additional burden for caregivers and family. (I am grateful to Grant Gillett for highlighting this point.)

35. It is, though, difficult to find published data to support this clinical impression, in part because in such circumstances life-sustaining treatment is usually withdrawn. See note 23, Wilkinson 2009.

36. ShewmonDA, HolmesGL, ByrnePA. Consciousness in congenitally decorticate children: Developmental vegetative state as self-fulfilling prophecy. Developmental Medicine and Child Neurology 1999;41(6):364–741040017010.1017/s0012162299000821; MerkerB. Consciousness without a cerebral cortex: A challenge for neuroscience and medicine. The Behavioural and Brain Sciences 2007;30(1):63–8110.1017/S0140525X0700089117475053, discussion at 134. Both of these papers note that, contrary to some claims, surviving children with hydranencephaly do respond to their surroundings and hence do not have features of a persistent vegetative state.

37. YamadaNK, KodnerIJ, BrownDE. When operating is considered futile: Difficult decisions in the neonatal intensive care unit. Surgery 2009;146(1):122–5.1954836510.1016/j.surg.2009.03.029

38. WilkinsonDJC, SavulescuJ. Knowing when to stop: Futility in the ICU. Current Opinion in Anesthesiology 2011;24(2):160–5.2129326710.1097/ACO.0b013e328343c5afPMC3252683

39. See note 38, Wilkinson, Savulescu 2011.

40. *King’s College Hospital NHS Foundation Trust v. T and Others* [2014] EWHC 3315 (Fam). ZT was born prematurely, but at 8 months of age suffered a cardiorespiratory arrest with subsequent evidence of “catastrophic irreversible hypoxic-ischemic injury to the brain.” At 17 months of age he remained in intensive care, dependent on mechanical ventilation. In addition, he had developed progressive severe hydrocephalus. Medical professionals sought permission from the court to withdraw mechanical ventilation from ZT (against the wishes of ZT’s family). The court subsequently granted permission, and ZT died. Bingham J. Boy taken off life support after judge rules there is no hope of a “miracle” dies. *Telegraph* 2014 6 Oct; available at http://www.telegraph.co.uk/news/health/news/11144708/Boy-taken-off-life-support-after-judge-rules-there-is-no-hope-of-a-miracle-dies.html (last accessed 3 Mar 2015).

41. See note 40, *King’s College NHS Trust v. T* 2014, at 9, 10, 12.

42. However, some authors have argued that consciousness and pain may be mediated by subcortical structures and have pointed to evidence that children with hydranencephaly do appear to respond to aversive situations. See note 36, Merker 2007.

43. See note 27, Wilkinson 2013, at 67–9. However, it should be noted that such infants may also have substantial reductions in terms of wider goods that they appreciate—that is, in the benefits that should be balanced against suffering. Accordingly, it is possible that even if infants suffer relatively little, the overall balance of benefits and burdens is tipped against treatment.

44. Complications of shunt insertion such as shunt infection or blockage may cause more suffering. One possibility is that shunt insertion itself would be in a child’s best interests but shunt revision (particularly if repeated) would not be.

45. WilkinsonD. Which newborn infants are too expensive to treat? Camosy and rationing in intensive care. Journal of Medical Ethics 2013;39(8):502–6.2335522910.1136/medethics-2012-100745

46. PhamAC, FanC, OwlerBK. Treating pediatric hydrocephalus in Australia: A 3-year hospital-based cost analysis and comparison with other studies. Journal of Neurosurgery. Pediatrics 2013;11(4):398–401.2339435710.3171/2013.1.PEDS12233 There have been no formal studies of the cost-effectiveness of surgery for hydrocephalus in developed countries; however, one study in sub-Saharan Africa found neurosurgical intervention to be highly cost-effective, costing approximately $100 per disability-adjusted life year averted. WarfBC, AlkireBC, BhaiS, HughesC, SchiffSJ, VincentJR, et al. Costs and benefits of neurosurgical intervention for infant hydrocephalus in sub-Saharan Africa. Journal of Neurosurgery Pediatrics 2011;8(5):509–21.2204437810.3171/2011.8.PEDS11163 (Note that this analysis looked at all cases of congenital hydrocephalus—i.e., not just SCH).

47. Based on costs of shunt insertion, revision, or infection from Pham et al. 2013 (see note 46) and rates of revision, infection, or mortality from Vinchon et al. 2012 (see note 13). Approximate cost per patient (USD): 11,000 + (2.7 × 8,000) + (0.38 × 66,000) = 57,680. Life years saved: 18 × (0.48 − 0.14) = 6.12. Cost per life year saved: 57,680 ÷ 6.12 = 9,424.84.

48. On conventional healthy utility scales, a life in full health is given a utility of 1, whereas death is given a utility of 0. States judged to be not worth living are rated negatively. A value of 0.1 has been applied to some very severely disabled states. One way of interpreting this is that someone would be prepared to exchange 10 years of life in such a state for 1 year in full health. Patrick DL, Starks HE, Cain KC, Uhlmann RF, Pearlman RA. Measuring preferences for health states worse than death. *Medical Decision Making* 1994;14(1):9–18. There are substantial challenges in assessing the quality of life of individuals who are unable to communicate, and in determining whether life with severe disability is an outcome that the individual would consider sufficiently beneficial to outweigh the harms of treatment. For the purposes of this article I am not endorsing a particular value for the quality of life of children with hydrocephalus (nor necessarily endorsing the QALY approach). The point here is to note that even if a very low (but positive) value is placed on survival, treatment may still be judged sufficiently cost-effective to justify providing it.

49. This value is close to (but lower than) the $100,000-per-QALY threshold that has sometimes been implicitly or explicitly endorsed. OwensDK. Interpretation of cost-effectiveness analyses. Journal of General Internal Medicine 1998;13(10):716–17.979882210.1046/j.1525-1497.1998.00211.xPMC1497852 Note that the Patient Protection and Affordable Care Act in the United States prohibited the use of cost-effectiveness thresholds in federal funding decisions. NeumannPJ, WeinsteinMC. Legislating against use of cost-effectiveness information. New England Journal of Medicine 2010;363(16):1495–7.2094266410.1056/NEJMp1007168 Here I am not endorsing the use of cost-per-QALY thresholds for neonatal treatment decisions. Rather, I am using them to indicate that if cost-effectiveness is assessed in the way that it is for other treatments in some parts of the world, surgery for SCH might be judged cost-effective.

50. MuraskasJ, ParsiK. The cost of saving the tiniest lives: NICUs versus prevention. Virtual Mentor 2008;10(10):655–8.2321180810.1001/virtualmentor.2008.10.10.pfor1-0810

51. This evidence might be taken to support arguments in favor of limiting intensive care (or may highlight the importance of providing adequate analgesia). Alternatively, the absence of evidence of pain perception might neutralize or at least reduce concerns about harm to the child from continued treatment.

52. Interpretation of functional imaging or tractography would be potentially extremely difficult in the context of the highly distorted cerebral architecture present in children with SCH. Of more practical concern is the fact that the very small numbers of surviving children would make it difficult to obtain sufficient data.

